# The Plant Sesquiterpene Nootkatone Efficiently Reduces *Heterodera schachtii* Parasitism by Activating Plant Defense

**DOI:** 10.3390/ijms21249627

**Published:** 2020-12-17

**Authors:** Samer S. Habash, Philipp P. Könen, Anita Loeschcke, Matthias Wüst, Karl-Erich Jaeger, Thomas Drepper, Florian M. W. Grundler, A. Sylvia S. Schleker

**Affiliations:** 1 Molecular Phytomedicine, Faculty of Agriculture, University of Bonn, Karlrobert-Kreiten-Straße 13, 53115 Bonn, Germany; grundler@uni-bonn.de; 2 Bioeconomy Science Center (BioSC), Forschungszentrum Jülich, 52425 Jülich, Germany; a.loeschcke@fz-juelich.de (A.L.); k.-e.jaeger@fz-juelich.de (K.-E.J.); t.drepper@fz-juelich.de (T.D.); 3 Chair of Food Chemistry, Institute of Nutritional and Food Sciences, Faculty of Agriculture, University of Bonn, Endenicher Allee 19C, 53115 Bonn, Germany; koenen@uni-bonn.de (P.P.K.); matthias.wuest@uni-bonn.de (M.W.); 4 Institute of Molecular Enzyme Technology, Faculty of Mathematics and Natural Sciences, Heinrich-Heine-University Düsseldorf, Forschungszentrum Jülich GmbH, 52426 Jülich, Germany; 5 Institute of Bio- and Geosciences IBG-1: Biotechnology, Forschungszentrum Jülich, 52425 Jülich, Germany

**Keywords:** plant natural product, terpenoid, plant parasitic nematode, beet cyst nematode, plant immune system

## Abstract

Plant parasitic nematodes, including the beet cyst nematode *Heterodera schachtii*, constitute a devastating problem for crops worldwide. The limited availability of sustainable management options illustrates the need for new eco-friendly control means. Plant metabolites represent an invaluable source of active compounds for the discovery of such novel antagonistic agents. Here, we evaluated the impact of eight plant terpenoids on the *H. schachtii* parasitism of *Arabidopsis thaliana*. None of the metabolites affected the plant development (5 or 10 ppm). Nootkatone decreased the number of adult nematodes on *A. thaliana* to 50%, with the female nematodes being smaller compared to the control. In contrast, three other terpenoids increased the parasitism and/or female size. We discovered that nootkatone considerably decreased the number of nematodes that penetrated *A. thaliana* roots, but neither affected the nematode viability or attraction to plant roots, nor triggered the production of plant reactive oxygen species or changed the plant’s sesquiterpene profile. However, we demonstrated that nootkatone led to a significant upregulation of defense-related genes involved in salicylic and jasmonic acid pathways. Our results indicate that nootkatone is a promising candidate to be developed into a novel plant protection agent acting as a stimulator of plant immunity against parasitic nematodes.

## 1. Introduction

Plant parasitic nematodes (PPNs) are a devastating problem to a wide range of crops worldwide [1]. Within PPNs, more than 4100 species have been described. The damage caused by PPNs was estimated to be more than 100 billion dollars per year worldwide, which is mainly caused by root knot nematodes (RKNs) and cyst nematodes (CNs) [2]. Among CNs, the beet cyst nematode *Heterodera schachtii* is a major problem and an important sedentary parasite of sugar beets, as well as more than 200 plant species belonging to 95 genera [3,4].

*H. schachtii* is a highly specialized obligate biotrophic nematode. When conditions are favorable, the infective second stage juveniles (J2s) hatch from eggs and spread in the soil. Having reached a host root, they enter it and migrate through the cells into the vascular cylinder. There, they search for a specific cell that is suitable to establish the initial syncytial cell (ISC) [5]. From the ISC, a highly active syncytial nurse cell system develops through fusion with the neighboring cells. The hypertrophic and hypermetabolic syncytiums serve as the only source of nutrients for the developing juveniles. The nematodes undergo three molts before reaching the adult stage. Males leave the root in a vermiform shape, whereas females remain at the root, grow to a lemon-like shape and, finally, rupture the root cortex. After mating, females produce eggs inside their bodies, until they eventually die. After death, their cuticles turn into brown-tanned cysts containing the eggs, which can survive for years until the conditions are again favorable [5].

For decades, the control of nematodes has heavily relied on chemical nematicides [6]. Nowadays, many of these “old” chemical nematicides are banned in most countries due to their environmental toxicity. Crop rotation and resistant or tolerant crop cultivars—if available for the specific crop plant and CN—have also been used and are still in use [7,8]. The effectiveness of crop rotation is limited though in some cropping systems because of the wide host range or long-term survival of CNs. Furthermore, the occurrence of virulent and resistance-breaking *H. schachtii* populations jeopardize the efficacy of using resistant crops [9]. All these factors minimize famers’ management options. Therefore, the urge for finding novel, environmentally friendly and effective management strategies to control nematodes is rising. One option is to make use of the enormous variety of plant metabolites that are available as bioactive compounds with diverse properties. The challenge is to identify their effects, modes of action and suitability for application in sustainable crop production.

Plant bioactive metabolites naturally play an important role in plant development and are involved in interactions with other organisms, such as insects, pathogens and neighboring plants [10,11,12,13,14,15,16]. Terpenoids are the largest and most diverse family of bioactive natural products, and several have allelopathic potential and are effective against different pathogens [12,16,17]. Multiple studies report the activity of terpenoids against nematodes [12,18,19]. For example, out of 22 investigated monoterpenes, borneol, carveol, citral, geraniol and α-terpineol were shown to be the most active against the RKN of *M. incognita*, as they decreased nematode hatching and survival, as well as the parasitism of *M. incognita* on tomato plants [12]. In another study, carvacrol, thymol, nerolidol, α-terpinene, geraniol, citronellol, farnesol, limonene, pseudoionone and eugenol were shown to have nematocidal activities against the nematode *Caenorhabditis elegans* [19]. Such reports demonstrate the potential of terpenoids as a source of nematicides. So far, no comparable studies were performed with a focus on *H. schachtii*. Therefore, we decided to investigate the effects of commercially available plant terpenoids against the CN *H. schachtii*.

In our study, we used as a selection of structurally diverse compounds the sesquiterpenes (−)-caryophyllene oxide, (+)-nootkatone and zerumbone and the triterpenes α-amyrin, β-amyrin and taraxasterol acetate, as well as apocarotenoid crocetin dialdehyde and the natural constituents of saffron stigma. Those metabolites occur in edible plants such as basil, citrus, ginger, tomato, dandelion and saffron. We investigated the effects of these eight terpenoids on *A. thaliana* plant growth, as well as against *H. schachtii*. We found nootkatone to significantly affect nematode parasitism and described the conceivable mode of action using several approaches. Exploring the tremendous range of natural compounds will help identifying molecules for novel environmentally friendly solutions in agricultural management strategies and expand our knowledge on interaction mechanisms.

## 2. Results

### 2.1. Effect of Plant Terpenoids on A. thaliana Growth

**Figure 1:**
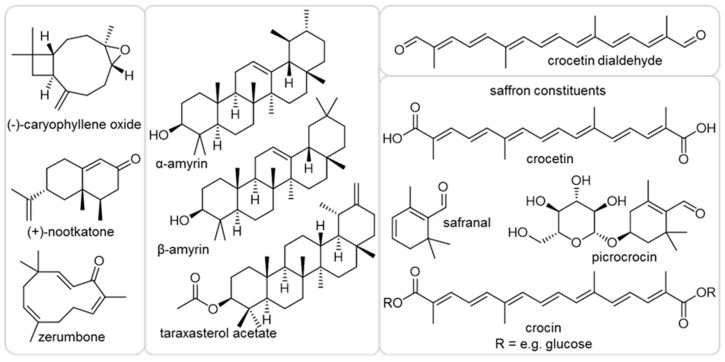
Chemical structures of the compounds used in this work.

**Figure 2:**
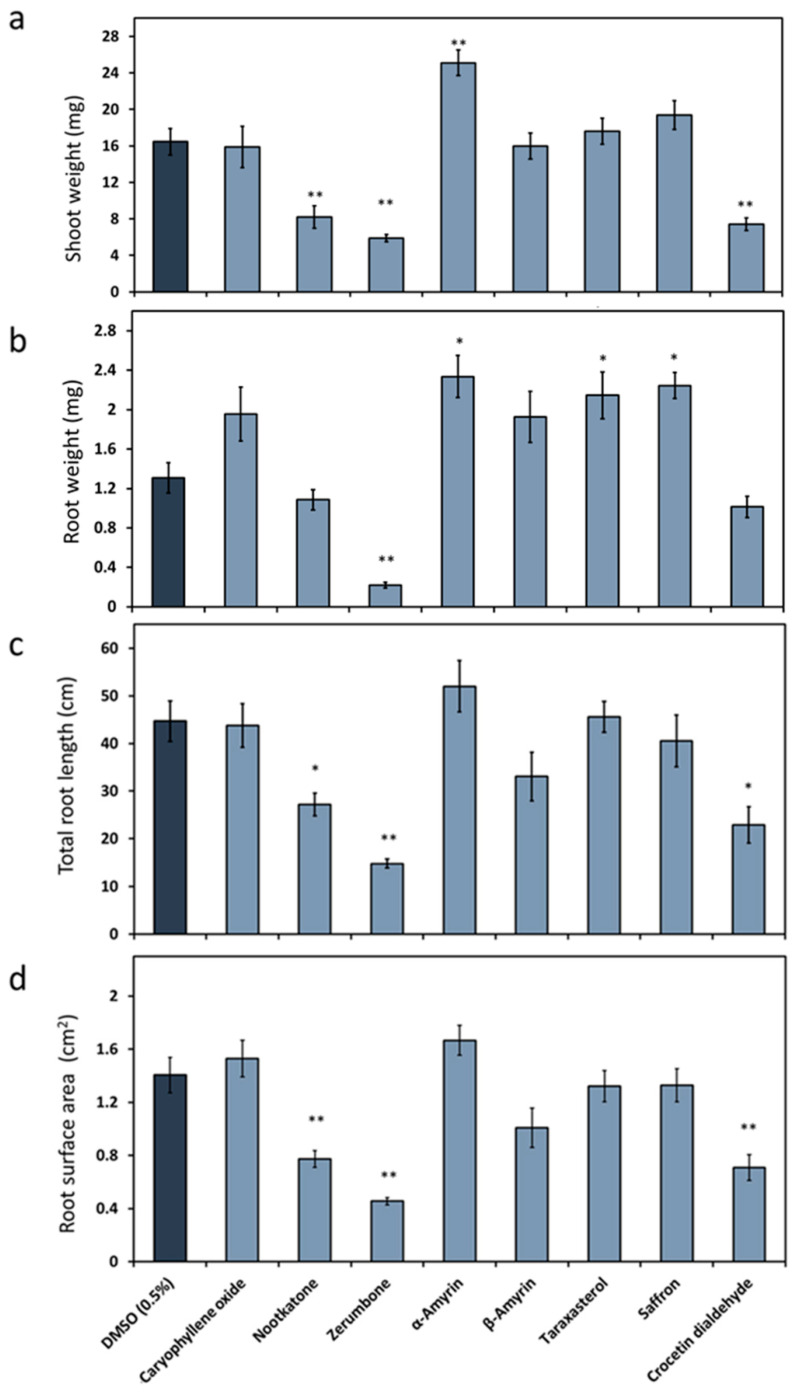
Effects of plant terpenoids on *Arabidopsis thaliana*. *A. thaliana* was cultured on a medium mixed with plant terpenoids (10 ppm, Figure 1) or dimethyl sulfoxide (DMSO) as the control. The following growth parameters were determined: (**a**) Average shoot weight (mg). (**b**) Average root weight (mg). (**c**) Average total length (cm). (**d**) Average root surface area (cm^2^). The data are based on three independent experiments. Each bar represents the mean ± SE of *n* = 12. The asterisk marks indicate significant differences to the control based on a *t*-test (* *p* < 0.05 and ** *p* < 0.01).

We first investigated the impact of the terpenoids (Figure 1) on *Arabidopsis* growth, as it is mandatory to work with a compound concentration that is neutral or positive for plant development. Therefore, the plant culturing medium was supplemented with 10 ppm of the chosen natural plant product. The effects varied between the metabolites (Figure 2). The shoot weight was significantly increased when the medium was mixed with α-amyrin, while it decreased in the presence of nootkatone, zerumbone or crocetin dialdehyde. No change was observed when caryophyllene oxide, β-amyrin, taraxasterol acetate and the natural saffron mixture were added to the medium. Only zerumbone reduced the plant root weight, while α-amyrin, taraxasterol acetate or the constituents of the saffron stigma had the opposite effect. The presence of nootkatone, zerumbone or crocetin dialdehyde decreased the total root length and surface area, while no significant effect was observed in presence of the other compounds. When *A. thaliana* was exposed to 5-ppm nootkatone, zerumbone and crocetin dialdehyde, no growth suppression of the plant could be observed anymore (data not shown). Based on these results, we decided to proceed with a 5-ppm final concentration of nootkatone, zerumbone and crocetin dialdehyde and with 10 ppm of the other terpenoids in the nematode infection assay experiment.

### 2.2. Effect of Plant Metabolites on H. schachtii Parasitism at A. thaliana

**Figure 3:**
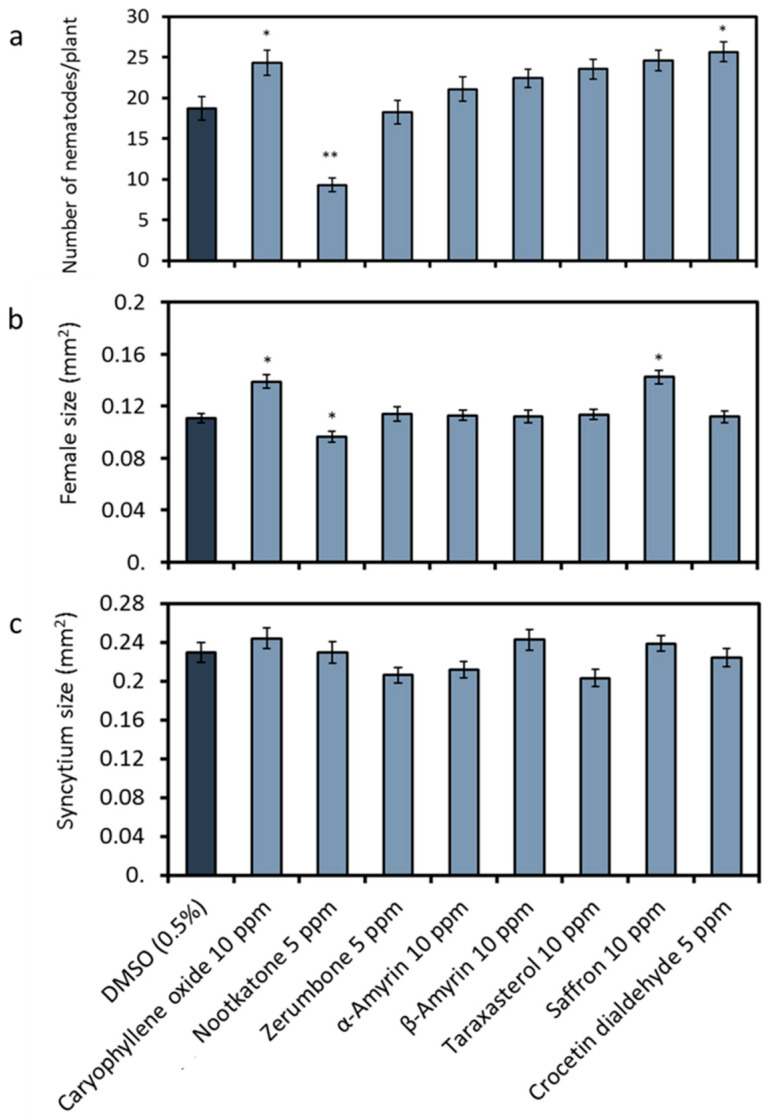
Effects of plant metabolites on *Heterodera schachtii* parasitism. *Arabidopsis* was grown on a medium complemented with plant terpenoids (Figure 1) or DMSO as the control and inoculated with *H. schachtii* J2s. Parameters representing the nematodes’ parasitic success were recorded: (**a**) Average number of total nematodes developed on an *Arabidopsis* Col-0 plant. (**b**) Average sizes of females at 13 days after inoculation (DAI). (**c**) Average sizes of syncytia at 13 DAI. Data are based on three independent experiments. Each bar represents the mean ± SE of *n* > 35. The asterisk marks indicate significant differences to the control based on a *t*-test (* *p* < 0.05 and ** *p* < 0.01).

In order to check the effects of the terpenoids on nematode parasitism, we infected the roots of *Arabidopsis* cultivated on a medium containing the metabolites with freshly hatched *H. schachtii* J2s. For evaluation, we determined the number of females and males at 12 days after inoculation (DAI) and measured the average sizes of females and their associated syncytia (Figure 3). Our analysis showed that nootkatone was the only terpenoid, which negatively affected nematode parasitism. Nootkatone reduced the average total number of mature nematodes per plant to 50% (Figure 3a). In contrast to this, caryophyllene oxide or crocetin dialdehyde led to an increase in the number of developed nematodes (Figure 3a). The average size of the females was slightly reduced only in the presence of nootkatone (87%) and was increased in the case of caryophyllene oxide (+25%) or the saffron extract (+28%) (Figure 3b). No effect was observed on the size of the associated syncytia in all treatments (Figure 3c).

### 2.3. Nootkatone Has No Direct Effect on J2s of H. schachtii

To explain the observed decrease of nematode parasitism at *A. thaliana*, we tested the direct effect of nootkatone on *H. schachtii* J2s. To do so, we treated the pre-infective J2s with several concentrations of nootkatone. We found that, after two days of incubation, the nematodes were vital and active even when exposed to 20 ppm of nootkatone (Figure S1). Thus, the nootkatone treatment had no observable effect on the J2s in comparison to the control.

### 2.4. Nootkatone Does Not Affect Nematode Attraction to the Plant but Inhibits Nematode Root Penetration

**Figure 4:**
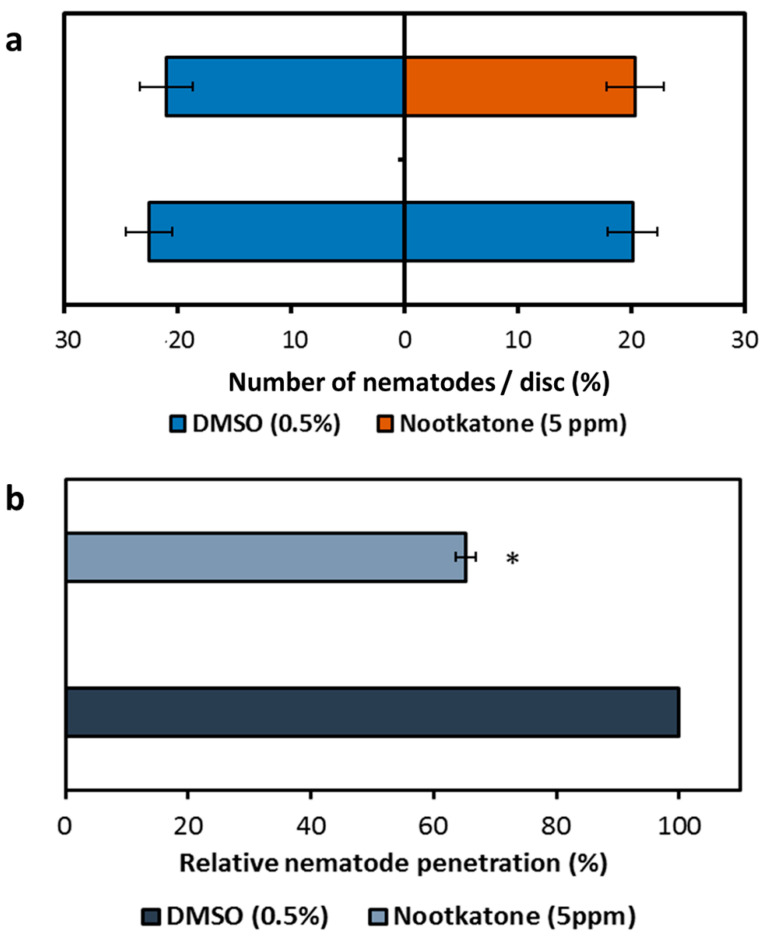
Effects of nootkatone presence in the growth medium on nematode behavior. (**a**) Chemotaxis analysis of *Heterodera schachtii* J2s towards *Arabidopsis thaliana* root-containing discs of Knop agar supplemented with nootkatone or DMSO. (**b**) Percentage of J2s’ penetrating roots of *A. thaliana* plants growing on Knop agar supplemented with nootkatone or DMSO. Values are means ± SE of 3 biological replicates, *n* = 27. The asterisk marks indicate significant differences to the control based on a *t*-test (*p* < 0.05).

Since the decrease of parasitism was not a result of the direct toxicity of nootkatone, the impact of the compound on nematode attraction towards and the penetration of *Arabidopsis* roots was determined. The presence of nootkatone in the growth medium did not affect nematode attraction to the plant, as there is no difference in the relative numbers of nematodes detected inside the test discs containing either nootkatone or dimethyl sulfoxide (DMSO). The same results were obtained in the case of a DMSO presence on both sides of the plates (Figure 4a).

Though no chemotaxis effects were observed, the percentage of J2s that successfully penetrated the *Arabidopsis* roots was about 40% decreased in the presence of nootkatone compared to the control (Figure 4b), revealing that the nootkatone-mediated inhibition of nematode parasitism functions at the stage of physical interaction between plant and nematode.

### 2.5. Nootkatone Activates Plant Defense

**Figure 5:**
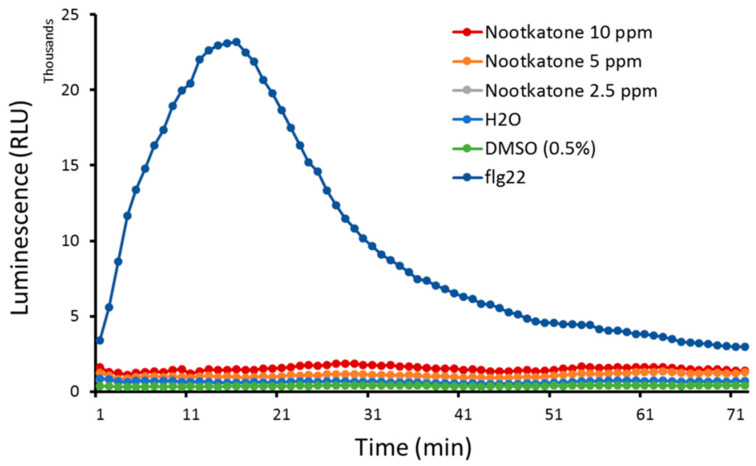
*Arabidopsis* leaf disc reactive oxygen species (ROS) production in response to nootkatone exposure. *A. thaliana* leaves were exposed to different concentrations of nootkatone, the bacterial elicitor peptide flg22 as a positive control or water and 0.5% DMSO as negative controls. The ROS presence was measured in relative light units (RLU) using a luminol-based assay during a 75-min-long incubation. Data show the mean value of 3 biological replicates, *n* = 12.

Since the inhibitory effects of nootkatone on *H. schachtii* parasitism were not a result of direct compound-induced nematode mortality, and we found that successful nematode root penetration events were significantly decreased in the presence of the metabolite, we decided to investigate whether nootkatone induces plant defense mechanisms. The reactive oxygen species (ROS) burst is one of the plant’s first defense lines. Therefore, we checked for changes in the ROS levels in *A. thaliana* leaf discs exposed to nootkatone, to the bacterial peptide flg22 as a positive control and to either 0.5% DMSO or water as the negative controls. As expected, the positive control flg22 induced a typical ROS burst peak. However, incubating leaf discs in nootkatone did not induce the ROS burst, as these samples showed a similar behavior to the negative controls (Figure 5).

**Figure 6:**
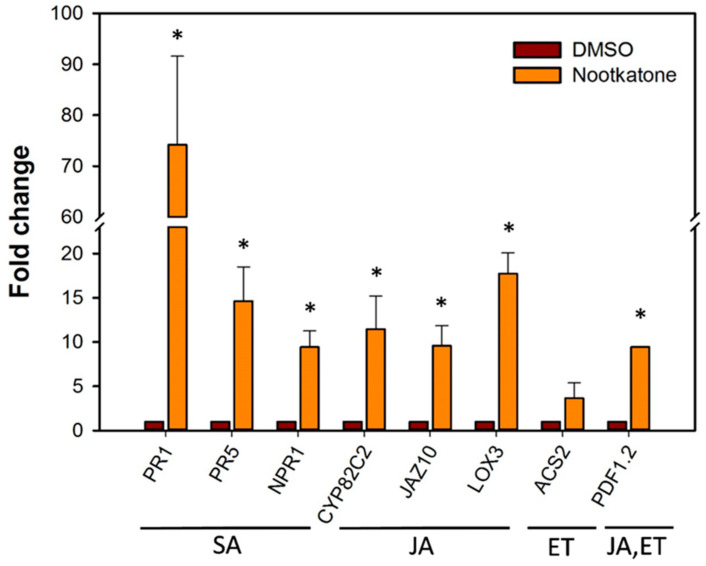
Relative mRNA levels of *Arabidopsis thaliana* defense-related genes upon nootkatone exposure quantified by using qRT-PCR. Fold change values were calculated and represent changes in mRNA levels in *Arabidopsis* plants cultivated on a medium mixed with 5-ppm nootkatone relative to that of *Arabidopsis* cultivated on a medium mixed with 0.5% DMSO as the control. Data are averages ± SE of three biologically independent experiments, each consisting of three technical replicates. *A. thaliana* β tubulin transcript was used as an internal control to normalize the data. The asterisk marks indicate significant differences based on a *t*-test (*p* < 0.05). SA: salicylic acid, JA: jasmonic acid and ET: ethylene.

To investigate further the lines of the plant defense, the transcript levels of several, commonly used marker genes involved in different plant defense hormone pathways, including salicylic acid (SA), jasmonic acid (JA) and ethylene (ET), were determined by quantitative real-time (qRT)-PCR (Table S1) [20]. The results showed that growing the plant on a medium mixed with nootkatone activated SA- and JA-related defense mechanisms. As shown in Figure 6, the transcript levels of genes related to SA were elevated up to 70-fold. The SA markers PR1 and PR5 were highly upregulated and reached a 74- and 14-fold change of transcript levels, respectively. The SA signaling gene NPR1 was nine-fold upregulated compared with the control. Similarly, the JA pathway was upregulated, as revealed by a transcript level increase of 10- and 11-fold for the biosynthesis genes JAZ10 and CYP82C2, respectively, and 20-fold for the signaling marker LOX3. The transcript level of the ET biosynthesis-related gene ASC2 showed no significant changes in the presence of nootkatone compared to the control (Figure 6).

### 2.6. Nootkatone Does Not Induce the Biosynthesis of Sesquiterpenes in A. thaliana

**Figure 7:**
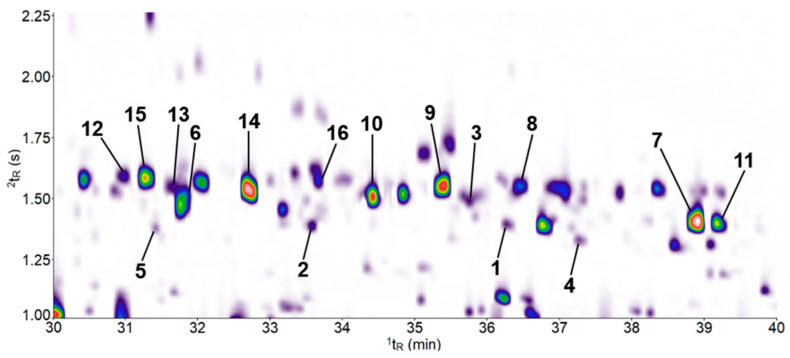
Contour plot of a headspace solid-phase microextraction (HS-SPME)–gas chromatography (GC)×GC–time-of-flight mass spectrometer (TOF–MS) chromatogram (TIC) demonstrating the separation of sesquiterpenes isolated from the headspace of a 30-day-old *Arabidopsis thaliana* plant grown on nootkatone-containing Knop agar. The ^1^tR (X-axis) corresponds to the retention time on the first dimension (^1^D) column and ^2^tR (Y-axis) to the retention time on the secondary (^2^D) column. The color gradient reflects the intensity of the TOF-MS signal on white background from low (violet) to high (red). Numbers at peaks refer to compound numbers, as defined in Table 1 and Figure S2. The 3D view of the chromatogram section shown can be found in Supplementary Figure S3.

In order to additionally investigate whether the nootkatone presence has an effect on the plant’s secondary metabolism of terpenoids, the volatile profile of *A. thaliana* was investigated using headspace solid-phase microextraction (HS-SPME)–gas chromatography (GC)×GC–time-of-flight mass spectrometer (TOF–MS). A total of 16 sesquiterpene hydrocarbons were found in the headspace of 30-day-old plants growing on both nootkatone-containing medium and the DMSO control (Table 1). The contour plot of the two-dimensional separation is shown in Figure 7 and the corresponding structural formulas of the sesquiterpenes in Figure S2. No differences in the sesquiterpene profiles were detected between the treatments. None of the compounds listed in Table 1 could be detected in blank tests with media-containing nootkatone or DMSO, except the sesquiterpene hydrocarbon nootkatene (7), which was already found in the medium containing nootkatone. The GC measurements performed indicated that nootkatone did not induce the formation of additional sesquiterpenes in *A. thaliana*.

## 3. Discussion

Secondary metabolites and mixtures such as essential oils are complex natural active agents formed by plants to be involved in several biological functions. Terpenoids are the major components of plant essential oils, and they are responsible for most of their biological properties [28,29,30]. Essential oils extracted from plants represent an invaluable source of bioactive compounds that were and still are used in pharmaceuticals, as well as in agrochemicals. Many of these essential oils and their main components are active against microbes, insects and nematodes. Particularly, terpenoids are very active and heavily studied components [12,13,14].

In this study, we tested several compounds belonging to the terpenoids for their activities against the nematode *H. schachtii*. We first evaluated the effects of caryophyllene oxide, nootkatone, zerumbone, α amyrin, β amyrin, taraxasterol acetate, saffron and crocetin dialdehyde (Figure 1) on *A. thaliana* to find a suitable concentration for the infection assay. Subsequently, we performed infection assays using an optimized, gnotobiotic system to study the interaction of *A. thaliana* and *H. schachtii* on Knop agar [31]. The ability of *H. schachtii* to infect the model host plant *A. thaliana* opens up a wide perspective of detailed studies of compounds against nematode parasitism [20,32]. Furthermore, we investigated the possible mode of action for the effective candidate terpenoid nootkatone.

The effect of terpenoids on plant growth is variable and has been reported in several studies to be related to the applied concentration. Therefore, testing the effect of the chosen compound concentration on the plant phenotype first was necessary. In our study, we found that *Arabidopsis* growth was affected in the presence of 10 ppm of nootkatone, zerumbone or crocetin dialdehyde in the growth medium. The inhibitory effect was abolished by decreasing the used concentration to 5 ppm. Similar observations were reported earlier using other terpenoids. In a previous study, four sesquiterpenoids, eight diterpenes and nine triterpenes were extracted from Tectona grandis and tested for phytotoxicity on several crop plants. Out of the 21 terpenes, 2 oxokovalenic acid and 19 hydroxyferruginol were phytotoxic. It was also shown that the toxicity was concentration-dependent and totally vanished for some terpenoids at low concentrations [17]. The same results were shown when trans-caryophyllene was tested against several plants, including *Arabidopsis*. The study showed that the effect was dependent on the plant species, as well as the tested concentration [15]. The study demonstrated that the effect is a result of changing the plant water status accompanied by oxidative damages [15].

Based on the results of the phytotoxicity tests, we conducted an infection assay with the plant parasite *H. schachtii* using the Knop medium containing 5 ppm of either nootkatone, zerumbone or crocetin dialdehyde or 10 ppm of the other terpenoids. Nootkatone was the only compound that decreased parasitism. There are no previous reports showing an effect of nootkatone against nematodes. However, many investigations demonstrated nootkatone activities against insects (for example, described in the previous studies [33,34,35,36]). On the other hand, caryophyllene was reported to be an attractant to nematodes in nature [37,38], which could explain the elevated parasitism of *H. schachtii* on *Arabidopsis* plants when exposed to this compound in our research.

Since we discovered nootkatone to remarkably inhibit *H. schachtii* parasitism, we further aimed to investigate the underlying mechanism. The mode of action of any antinematode compound can be categorized as (1) direct toxicity or repellent of nematodes or (2) indirect effects by activating plant defense mechanisms against nematodes. Our results revealed no lethal effect of nootkatone against nematodes even after two days of exposure. Therefore, we concluded that the effect was not direct. In contrast, previous studies demonstrated the direct effect of nootkatone against some arthropods—specifically, fleas, ticks and mosquitos—but still with an unclear mode of action [39]. To determine the mechanism against mosquitos, McAllister and Adams (2010) challenged mutant strains of *Anopheles gambiae* Giles harboring mutations at three different target sites known to mediate an insecticide resistance with nootkatone and compared the lethal dosages. The altered target sites evaluated were the sodium channel para-locus mutation (L1014 F KDR) that confers permethrin resistance, the ACE-1 gene that confers organophosphate and carbamate resistance and a γ aminobutyric acid receptor mutation of the Rdl locus conferring dieldrin resistance. In the study, they found that the *A. gambiae* wild type and all three mutants were equally sensitive to nootkatone, as there were no significant differences in the required lethal doses determined. Therefore, they concluded that nootkatone acts through a different mode of action than existing chemicals currently used in mosquito control.

As nootkatone had no nematocidal effect, we decided to analyze if nootkatone affects the nematode’s behavior by causing positive or negative chemotaxis. Our results displayed that the presence of nootkatone in the medium did not affect the J2s’ preference for the plant. We further demonstrated that the nematodes were equally attracted to both sides of the treatment in presence and absence of nootkatone. This means that nootkatone does not affect the nematode’s orientation towards plants. However, the J2 penetration percentage of the plant roots was significantly decreased in the presence of nootkatone compared with the untreated control. This indicates that the plant defense was activated and inhibited J2 penetration and nematode development. To analyze the molecular changes nootkatone induces in the plant, we first determined whether the compound triggers a ROS burst. The production of ROS is one of the earliest defense responses observed in various plant–pathogen interactions [40,41]. Additionally, the elevation of plant ROS plays a major role in plant resistance against nematodes [42]. Therefore, we measured the presence of ROS upon the exposure to several concentrations of nootkatone. Plant ROS levels were not elevated in nootkatone-treated leaf discs, demonstrating that nootkatone is functioning through another mechanism.

Another defense option is the emission of volatile organic compounds (VOCs) that protect plants against herbivores and pathogen attacks. Sesquiterpenes, as a group of the emitted VOCs, have been shown to play a major role in the plant defense, including direct activity against pathogens (e.g., inducing oxidative stress) or triggering defense signaling pathways [16,43]. Therefore, we also measured the plant sesquiterpene profile upon exposure to nootkatone to determine if the presence of nootkatone induces the emission of sesquiterpenes, which, in return, could play a role against nematodes. We performed a GC analysis of the volatile profile of *A. thaliana* plants cultivated on a nootkatone-containing medium and compared it with the profile of the plants cultivated on a medium with DMSO as the control. Our results revealed no detectable differences between the treatments. The measured volatile profiles overlap with the results from previous studies, where terpene profiles of six-week-old plants were analyzed [21,44]. In addition, the sesquiterpenes β-cedrene, β-barbatene, 4-epi-α-acoradiene and α-curcumene were found in our analysis. With these results, we concluded that nootkatone is not acting through activating the emission of additional plant sesquiterpenes. Whether the emitted quantity of the individual sesquiterpenes differs between the control and the nootkatone-treated plant could not be determined, as the conducted analysis is purely qualitative.

Moreover, we examined whether defense mechanisms involving the SA, JA and ET pathways are triggered by nootkatone by examining the transcript levels of several marker genes. We observed that the expression of several SA and JA marker genes were increased in the presence of nootkatone. This explains the reduced parasitism of *H. schachtii* in our infection assay, as these results are in agreement with previous studies reporting that elevated concentrations of SA and JA negatively affected the parasitism of *H. schachtii* [20,45]. Furthermore, it was shown that an increased ET level attracts nematodes towards the plant [20]. Our results revealed that ET biosynthesis was not affected by the presence of nootkatone, which could explain why nootkatone did not change the nematodes’ chemotactic behavior.

The presented results clearly demonstrate for the first time that nootkatone reduces *H. schachtii* parasitism on its host plant *Arabidopsis* and that this is caused by an indirect mode of action involving the plant. One way to explain this effect is the activation of the plant defense through elevating the expression of genes involved in the biosynthesis of the SA and JA genes upon nootkatone exposure. The molecular basis of how nootkatone interacts with the plant and initiates the observed effects still requires elucidation. Analogous to the observations by Pretel et al. (2019) for insects, it would moreover be interesting to determine whether and how the derivatization of nootkatone changes its activity and efficacy against nematodes. This will be an important factor to consider in terms of nootkatone formulation for field applications. One approach to increase the molecule’s solubility in water, as well as its photo- and thermal stability, but ensuring its permanent release to the environment, is encapsulation in cyclodextrins [46]. In view of a possible application of nootkatone in nematode management strategies for plant protection, this is one among many open questions to be clarified. Knowledge about the suitable application strategy, the necessary exposure time and time point, as well as the physical and biochemical characteristics of nootkatone in the field in relation to the compound efficacy, will guide the way to product design and practical use.

Taken together, our results present nootkatone as a promising natural compound and potentially environmentally safe option to manage nematodes in agricultural cropping systems. This is supported by the fact that nootkatone is naturally produced by, e.g., citrus fruits (it is a major component of the grapefruit aroma), and recognized as nontoxic to humans, that the compound is approved by the Food and Drug Administration (FDA) as a food additive and that nootkatone is already used as a citrus flavor and fragrance ingredient of commercial products. Moreover, it is considered as an environmentally friendly pesticide against mosquitos and ticks and does not persist in the environment [47]. Beside the possibility of extracting nootkatone from plants, it can alternatively be obtained by a recombinant microbial production. Recently, *Rhodobacter capsulatus* was introduced as a phototrophic platform organism for synthesizing sesquiterpenoids, which could also facilitate the production process [48]. High commercial interest with regards to nootkatone applications is currently rising, as demonstrated by according the biotechnological production lines. Future investigations to obtain a detailed understanding of how nootkatone activates the plant defense to optimize a suitable compound formulation and to perform soil and field application studies are necessary to evaluate the compound’s applicability in agricultural nematode management strategies.

## 4. Materials and Methods

### 4.1. Chemicals

The following plant metabolites used in our study were ordered: (−)-caryophyllene oxide, (+)-nootkatone, zerumbone, β-amyrin, crocetin dialdehyde, saffron stigma (Sigma-Aldrich, St. Louis, MO, USA), α amyrin (Carl Roth, Karlsruhe, Germany) and taraxasterol acetate (Biorbyt, Cambridge, UK) (Figure 1). All compounds were dissolved in dimethyl sulfoxide (DMSO) in order to be used in the assays. Saffron threads were incubated in DMSO for 1 h on a rotary shaker (100 rpm); then, the supernatant was collected and used in the work. A C_7_-C_30_ saturated alkanes standard solution was also obtained from Sigma-Aldrich to calculate the GC retention indices of the sesquiterpenes formed by *A. thaliana*. The liquid standards used were β-elemene, ordered from Abcam plc (Cambridge, UK) and (−)-trans-caryophyllene (sum of enantiomers, purity: ≥98.0%) and (−)-α-cedrene (sum of enantiomers, purity: 96.4%) purchased from Sigma-Aldrich.

### 4.2. Nematode Culture

The CN *H. schachtii* Schmidt was reared on white mustard (*Sinapis alba* L. cv. Albatros), which was cultivated aseptically on agar medium supplemented with 0.2% Knop’s nutrient solutions under long day conditions with 16-h light and 8-h dark at 25 °C. Mature cysts were collected around 2 months after inoculation in funnels and hatched in 3-mM ZnCl_2_ [31]. The hatched preparasitic J2s were collected and used in the infection and mortality assays.

### 4.3. Effect of Terpenoids on A. thaliana Growth

In order to investigate the effect of the plant metabolites on *Arabidopsis* growth, plants of the ecotype Col-0 were grown aseptically on agar medium supplemented with modified 0.2% Knop’s nutrient solutions and a final concentration of 10 ppm of the compounds under long day conditions with 16-h light and 8-h dark at 25 °C, as described previously [31]. Medium containing 0.5% DMSO was used as control. After 15 days of growth, fresh weight of root and shoot was measured, and root surface area, total root length and root diameter were evaluated using Epson Perfection V700 Photo root scanner and analyzed using WinRHIZO software (Regent Instruments, Québec, QC, Canada). Three independent biological experiments, each consisting of four technical replicates, were performed. Data was statistically analyzed using *t*-test at *p*-value < 0.5.

### 4.4. Infection Assay

To study the effect of the plant metabolites on nematode parasitism, an in vitro infection assay was performed using *A. thaliana* ecotype Col-0 plants grown on 0.2% Knop’s agar medium supplemented with plant terpenoids (5 or 10-ppm final concentration). Plants were grown aseptically with a light period of 16 h and 8-h dark at 25 °C, as described previously [31] The infection assay was performed as described previously [49]. Briefly, roots of 10-day-old seedlings were inoculated with 60–70 J2s per plant. On the 12th day after inoculation (DAI), numbers of adult males and females were counted. Sizes of females and associated syncytia were measured 13 DAI using Leica M165C Binoculars (Leica Microsystems, Wetzlar, Germany) and Leica Application Suite software. Experiments were repeated 3 times and analyzed using a *t*-test. Each experiment consisted of 12 plants per treatment.

### 4.5. Attraction Assay

*Arabidopsis* plants were grown on Knop’s agar medium mixed with nootkatone (5-ppm final concentration) or DMSO (0.5%) as the control for 10 days, as described above. From these plates, agar discs (1-cm diameter) containing roots were cut to use in the chemotaxis assay. For the chemotaxis assay, Petri plates were filled with 0.2% Knop’s agar medium, and two agar discs (1-cm diameter) per plate were removed to obtain two opposing holes with 2-cm distance to each other. Into these holes, the discs containing the roots were placed in the following two settings to test nematode attraction: For the first variant, the disc on one side contained roots in nootkatone-supplemented agar, and the disc on the other side contained roots in DMSO-supplemented agar. In the second treatment, both removed discs were replaced with agar discs containing roots in DMSO-supplemented agar. Subsequently, around 70 *H. schachtii* J2s were placed exactly in the middle between the opposing discs and incubated at 25 °C. After 8 h, the number of J2s inside each disc was counted using a dissecting microscope, and the percentage of attracted J2s was calculated. Experiments were repeated 3 times, each experiment consisted of 6 replicates. Data was analyzed using a *t*-test.

### 4.6. Root Penetration Assay

To determine the effect of nootkatone on the ability of J2s to penetrate *A. thaliana* roots, 10-day-old *Arabidopsis* plants grown on 0.2% Knop’s agar medium mixed with nootkatone (5 ppm) were inoculated with 60–70 freshly hatched *H. schachtii* J2s. After 2 days, the number of nematodes that successfully penetrated the roots was documented. *Arabidopsis* plants grown on 0.2% Knop’s agar medium mixed with DMSO (0.5%) and infected with J2s served as a control for comparison. Experiments were repeated 3 times; each experiment consisted of 6 replicates. Data was analyzed using a *t*-test.

### 4.7. Mortality Assay

The direct effect of nootkatone on nematode viability was investigated by using *H. schachtii* J2s. The mortality experiment was conducted in 96-well plates (Greiner Bio-One, Solingen, Germany) under aseptic conditions. Each well contained 60 μL of the test solution (5, 10 or 20-ppm nootkatone dissolved in 0.5% DMSO or 0.5% DMSO as the control) and around 100 nematodes. Plates were incubated for 2 days at 25 °C in the dark. Numbers of living (moving) and dead (not moving) nematodes were evaluated using a dissecting microscope and 1-mM sodium hydroxide to provoke the nematode activity of living J2s. Percentage of motile nematodes was calculated. The experiment was set up in 3 biological replications (each contains 3 wells per concentration). Data was collected and statistically analyzed for significance using a *t*-test at *p*-value < 0.5.

### 4.8. Reactive Oxygen Species (ROS) Measurements

In order to check if the presence of nootkatone activates an ROS-mediated plant defense, an ROS burst was evaluated using a luminol-based assay with leaf discs and a 96-well plate luminometer (Tecan infinite 200Pro; Tecan Group Ltd., Männedorf, Switzerland), as described previously [50]. Briefly, leaf discs of 14-day-old *Arabidopsis* plants were incubated overnight in sterile double-distilled water, then transferred to cavities of a 96-well plate filled with 5, 10 or 20-ppm nootkatone or with 0.5% of DMSO or with sterilized double-distilled water as negative controls or with the bacterial peptide flg22 (1 µM) as a positive control [50]. Light emission was measured in relative units over a 75-min-long incubation period and analyzed using the Tecan i-control software. Data was tabulated and plotted. Four leaf discs were used per treatment as technical replicates. The experiment was repeated 3 times.

### 4.9. Quantitative Real-Time PCR

The transcript level of several marker and signaling genes involved in salicylic acid (SA), jasmonic acid (JA) and ethylene (ET) pathways (Table S1) was measured to investigate the effect of nootkatone on a hormone-mediated plant defense [20]. Therefore, *Arabidopsis* plants were grown for 13 days on Knop medium mixed either with 5-ppm nootkatone or with 0.5% DMSO as a control. Subsequently, plants were collected, RNA was extracted using a NucleoSpin RNA kit (Macherey-Nagel, Düren, Germany), including DNase treatment, and converted to cDNA using the High-Capacity cDNA Reverse-Transcription Kit (Applied Biosystems, Foster City, CA, USA) and oligo-dT primer following the manufacturers’ protocols. The expression of the genes was determined using the obtained cDNAs and the StepOnePlus Real-Time PCR System (Applied Biosystems, Foster City, CA, USA) at the following conditions for amplification: 95 °C for 15 s and 60 °C for 30 s (40 cycles). Within each sample, 10 μL of Fast SYBR Green qPCR Master Mix (Invitrogen, Carlsbad, CA, USA), 9 μL of the specific primer mixture and 1 μL of cDNA were used. The amplification data was analyzed using the StepOnePlus Real-Time PCR software (Thermo Fisher, Waltham, MA, USA) to create C_t_ (threshold cycle) values. The generated data was analyzed and fold expression was calculated, as described previously [51]. Transcript of the β tubulin gene was used as an internal control for all experiments (Table S1). Experiments were performed in three technical replicates and independently repeated three times. Data was statistically analyzed using *t*-test.

### 4.10. HS–SPME–GC×GC–TOF–MS Analysis

The sesquiterpene profile of 30-day-old *A. thaliana* grown on a nootkatone-containing medium was analyzed by multidimensional gas chromatography (GC) coupled with a time-of-flight mass spectrometer (TOF–MS). All plants were grown directly in 20-mL headspace vials to avoid a loss of volatiles. A CTC Combi PAL-xt autosampler (CTC Analytics, Zwingen, BL, Switzerland, CHE) with a heatable agitator and a solid-phase microextraction (SPME) fiber-conditioning station was used to extract the volatiles emitted by *A. thaliana* from the headspace. The SPME extraction conditions were based on a method previously described [52]. The samples were first heated at 45 °C for 10 min without stirring. Subsequently, a divinylbenzene/carboxen/polydimethylsiloxane (DVB/CAR/PDMS, 50/30 µm, Stableflex (2 cm) 24Ga) SPME fiber from Supelco (Bellefonte, PA, USA) was exposed in the headspace of *A. thaliana* for 30 min. The volatiles were desorbed in the GC inlet at 250 °C for 5 min. After each desorption process, the fiber was reconditioned at 250 °C for 15 min to avoid carryover. In addition, an empty headspace vial was measured between each sample as a control, and no sample carryover was detected. Extraction at room temperature directly from the tray produced the same qualitative results but with lower peak intensities.

A 7890B gas chromatograph (Agilent Technologies, Bellefonte, PA, USA) interfaced with a Zoex ZX2 GC×GC cryogenic modulator (Zoex Corp., Houston, TX, USA) and a Bench TOF-Select™ time-of-flight mass spectrometer (Markes International Limited, Llantrisant, Wales, UK) were used to perform the GC×GC–TOF–MS measurements. The instrument was fitted with a two-dimensional column set consisting of a highly polar DB-WAX ultra-inert (poly(ethylene glycol); 30 m × 0.25 mm × 0.25 µm (length × internal diameter × film thickness); Agilent Technologies, Bellefonte, PA, USA) as the first dimension (^1^D) column and a moderately polar MEGA-17 MS FAST (poly(50%-phenyl-50%-methylsiloxane); 1.7 m × 0.10 mm × 0.10 µm; MEGA s.n.c., Legnano, Milan, Italy) as the second dimension (^2^D) column. Helium was used as carrier gas at a constant flow rate of 1 mL min^−1^. The ^1^D column was initiated at 35 °C (5 min hold), then heated at 5 °C min^−1^ to 120 °C (0-min hold) and, finally, to 220 °C at 3 °C min^−1^ (5 min hold). The modulator was heated with a +25 °C offset. The modulation time was 5 s with a 350-ms pulse length. The MS transfer line and ion source were maintained at 250 °C. Mass spectrometric parameters were as follows: ionization energy −70 eV, scan speed 100 Hz, recorded mass range 35–250 *m/z* and detector voltage −2222 V. GC×GC–TOF–MS data were collected in ProtoTof (Markes International Limited, Llantrisant, Wales, UK; version 2.0) and processed with ChromSpace (Markes International Limited, Llantrisant, Wales, UK; version 1.5). Data was evaluated as a contour plot of the total ion chromatograms (TIC) obtained in the first (^1^D) and secondary (^2^D) dimensions. Analytes were identified based on matching the mass spectrum to a library spectrum, similarity in a retention index and comparison to available authentic reference compounds. The results obtained were confirmed by measuring 3 biological replicates and 3 technical replicates each.

## Figures and Tables

**Table 1:** Sesquiterpene hydrocarbons identified in the headspace of 30-day-old *Arabidopsis thaliana*.

No.	Compound ^a^	*I* ^b^	*I* (lit.) ^c^	Identification ^d^
12	α-cedrene	1586	1582 [21]	ms (936, 942), ri, std
15	α-barbatene	1596	1572 [21]	ms (844, 864), ri
5	β-elemene	1600	1605 [22]	ms (876, 876), ri, std
13	β-cedrene	1607	1599 [23]	ms (820, 834), ri
6	(*E*)-β-caryophyllene	1613	1619 [22]	ms (909, 913), ri, std
14	*cis*-thujopsene	1640	1620 [24]	ms (924, 927), ri
2	(*E*)-β-farnesene	1666	1666 [25]	ms (913, 923), ri
16	β-barbatene	1669	n.a. ^e^	ms (874, 876)
10	4-epi-α-acoradiene	1692	n.a. ^e^	ms (885, 895)
9	β-chamigrene	1722	1740 [26]	ms (888, 905), ri
3	β-bisabolene *	1734	1729 [21]	ms (889, 893), ri
1	(*E*,*E*)-α-farnesene *	1750	1747 [27]	ms (902, 922), ri
8	α-chamigrene	1755	1753 [21]	ms (845, 894), ri
4	α-curcumene	1780	1780 [22]	ms (876, 892), ri
7	Nootkatene	1829	1815 [24]	ms (872, 887), ri
11	Cuparene	1838	1825 [24]	ms (906, 915), ri

^a^ Unidentified compounds are not listed. ^b^ Retention index *I* on a DB-WAX ultra-inert column. ^c^ Retention index data from the literature. ^d^ Compound identification is based on matching the mass spectrum to a library spectrum (ms, match factor and reverse match factor given in brackets) identical or closely matching the retention index (ri) and a comparison to a commercially available standard compound (std). ^e^ Retention index data on a WAX column were not available. * Only detectable in two out of three biological replicas. The recorded mass spectra of the listed sesquiterpene hydrocarbons can be found in Supplementary Table S2.

## Supplementary Materials

Supplementary materials can be found at https://www.mdpi.com/1422-0067/21/24/9627/s1: Figure S1. In vitro effect of nootkatone on mortality of *Heterodera schachtii* J2s, Figure S2. Sesquiterpene hydrocarbons found in the headspace of a 30 days old *Arabidopsis thaliana* plant, Figure S3. Surface chart (TIC) of a HS–SPME–GC×GC–TOF–MS measurement of sesquiterpene hydrocarbons of a 30 days old *Arabidopsis thaliana*, Table S1. Primers used in the study, Table S2. Recorded mass spectra of the identified sesquiterpene hydrocarbons.
