# Supplementary material for: The Plant Sesquiterpene Nootkatone Efficiently Reduces Heterodera schachtii Parasitism by Activating Plant Defense
Source: Int J Mol Sci. 2020 Dec 17;21(24):9627. doi: 10.3390/ijms21249627 (PMC7765886; doi:10.3390/ijms21249627)
Supplement: Supplementary file 1 [file ijms-21-09627-s001.pdf]

International Journal of *Molecular Sciences*

MDPI

Supplementary Data

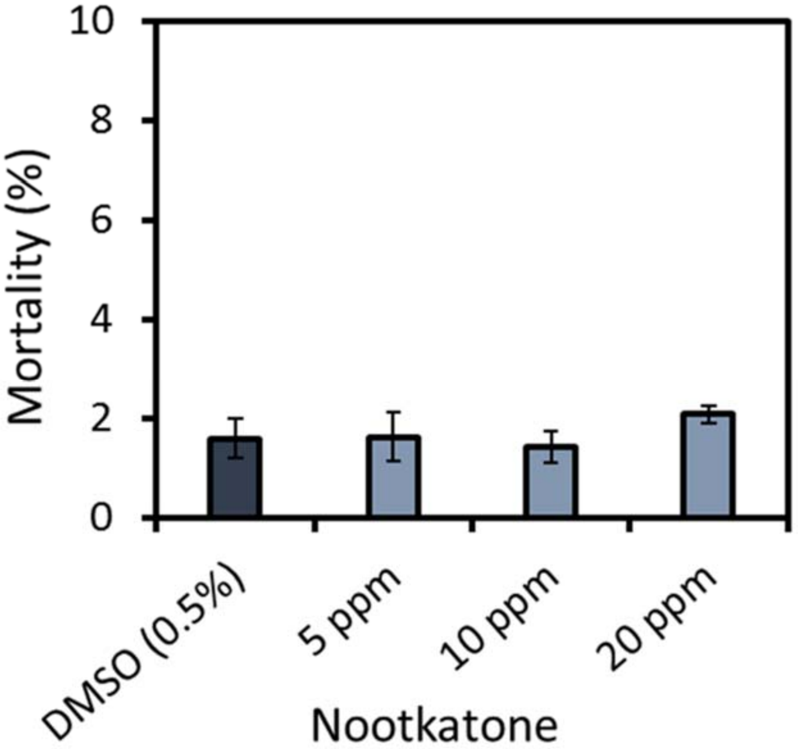

Figure S1. In vitro effect of nootkatone on the mortality of *Heterodera schachtii* J2s. *H. schachtii* J2s were incubated in different concentrations of nootkatone for two days. Values are means ± SE of three biological replicates, *n* = 12 (around 100 nematodes each).

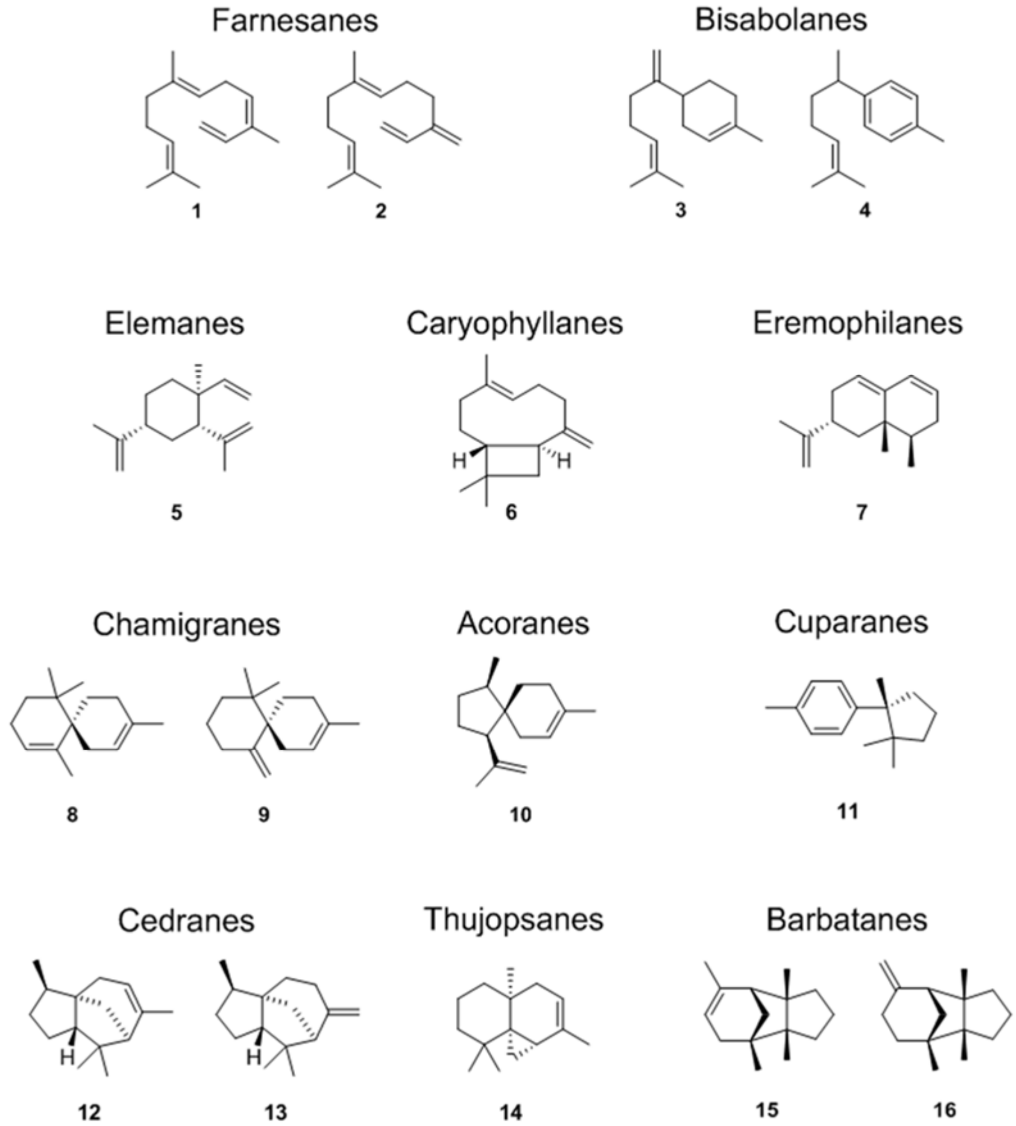

Figure S2. Sesquiterpene hydrocarbons found in the headspace of a 30-day-old *Arabidopsis thaliana* plant. 1 and 2, acyclic sesquiterpenes; 3–5, monocyclic sesquiterpenes; 6–11, bicyclic sesquiterpenes; 12–16, tricyclic sesquiterpenes and 4 and 11, aromatic sesquiterpenes. The identified compounds are grouped according to the type of sesquiterpene skeleton.

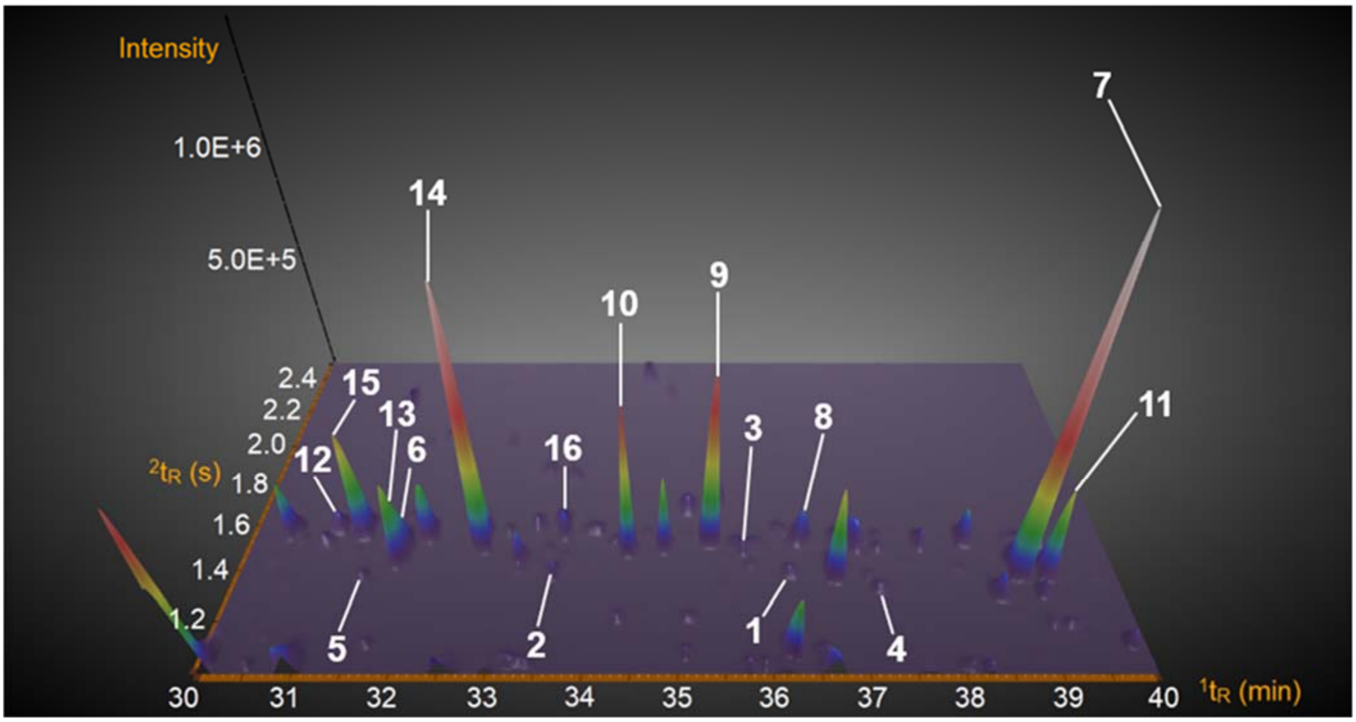

Figure S3. Surface chart (TIC) of a HS–SPME–GC×GC–TOF–MS measurement of sesquiterpene hydrocarbons of a 30-day-old *Arabidopsis thaliana*. The data is displayed in 3D format, so that the signal intensities are represented in the form of conical signals.

Table S1. Primers used in the study.

*Gene	Gene Accession	Gene Correspondence	Primer (5′→3′)
Tubulin 4	at5g44340	Internal control	F: TTTCCGTACCCTCAAGCTCG<br>R: GTGAAGCCTTGGGAATGGGA
PR1	at2g14610	SA markers	F: AAGGAGCATCATATGCAGGA<br>R: ATTTAAATAGATTCTCGTAATCTCAGC
PR5	at1g75040	SA markers	F: AGGCTGCAACTTTGACGC<br>R: AGAAATCTTTGCCGCCATC
NPR1	at1g64280	SA signaling	F: AAACCGAGTTGCACTTGCTC<br>R: AGTGATGTCCGCTTTTCACC
CYP82C2	at4g31970	SA markers	F: GTGAAAGCACTAGGCGAAGC<br>R: ATCCGTTCCAGCTAGCATCA
JAZ10	at5g13220	JA biosynthesis	F: TCGCAAGGAGAAAGTCACTGCAAC<br>R: CGATTTAGCAACGACGAAGAAGGC
LOX3	at1g17420	JA signaling	F: CGGATAGAGAAAGAGATTGAGAAAAGGAAC<br>R: AGGTACACCTCTACACGTAACACCAGGC
ACS2	at1g01480	ET biosynthesis	F: GGATGGTTTAGGATTTGCTTTG<br>R: GCACTCTTGTTCTGGATTACCTG
PDF1.2	at5g44420	JA, ET responsive gene	F: TGTTCTCTTTGCTGCTTTCG<br>R: TTTCCGCAAACCCCTGAC

* PR1, PATHOGENESIS-RELATED GENE 1; PR5, PATHOGENESIS-RELATED GENE 5; NPR1, NONEXPRESSER OF PR GENES 1; CYP82C2, CYTOCHROME P450, FAMILY 82, SUBFAMILY C, POLYPEPTIDE 2; JAZ10, JASMONATE-ZIM-DOMAIN PROTEIN 10; LOX3, LIPOXYGENASE 3; ACS2, 1-AMINO-CYCLOPROPANE-1-CARBOXYLATE SYNTHASE 2 and PDF1.2, PLANT DEFENSIN 1.2.

Table S2. Recorded mass spectra of the identified sesquiterpene hydrocarbons.

No.	Compound	Mass Spectrum
**12**	α-cedrene	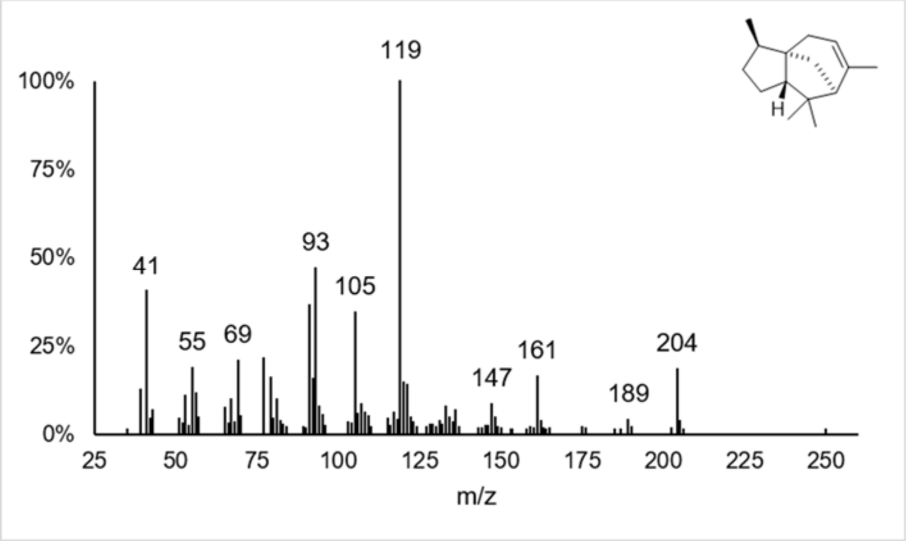

**15**	α-barbatene	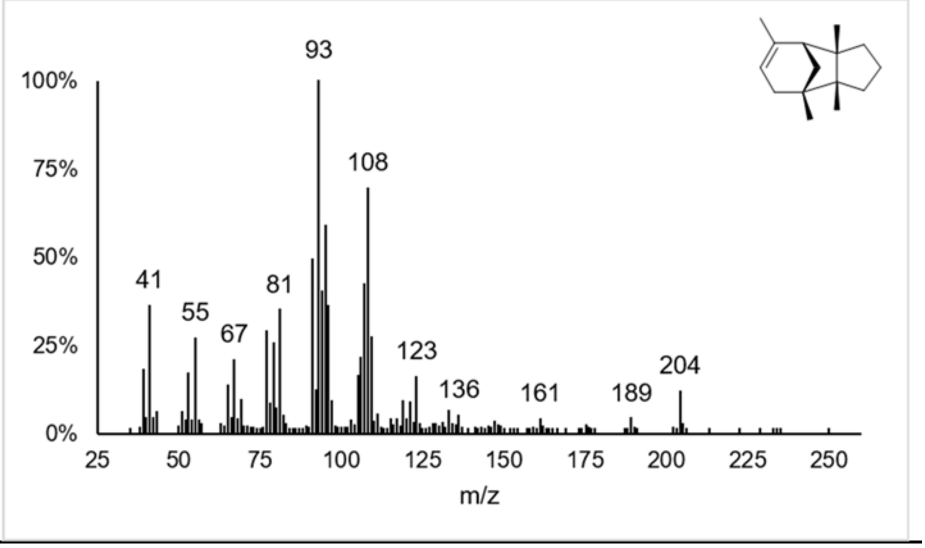

Table S2. *Cont.*

No.	Compound	Mass Spectrum
**5**	β-elemene	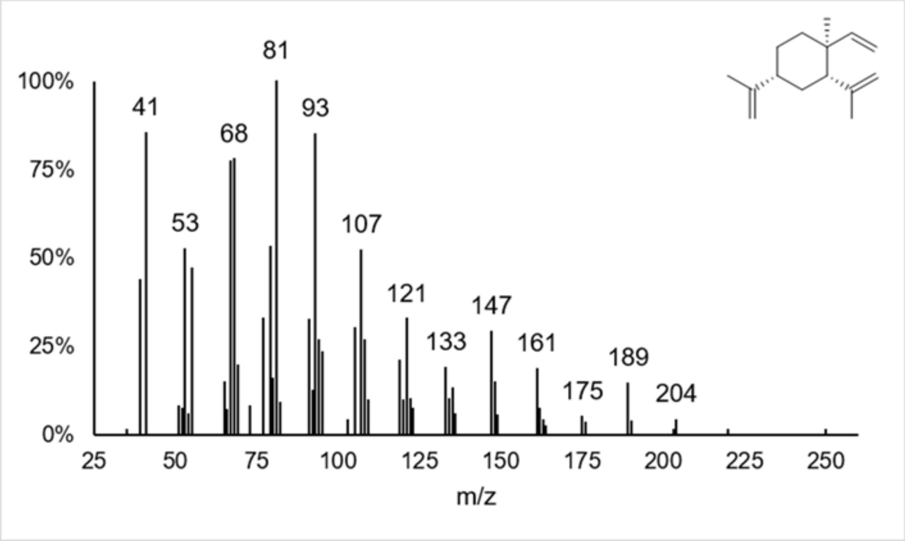
**13**	β-cedrene	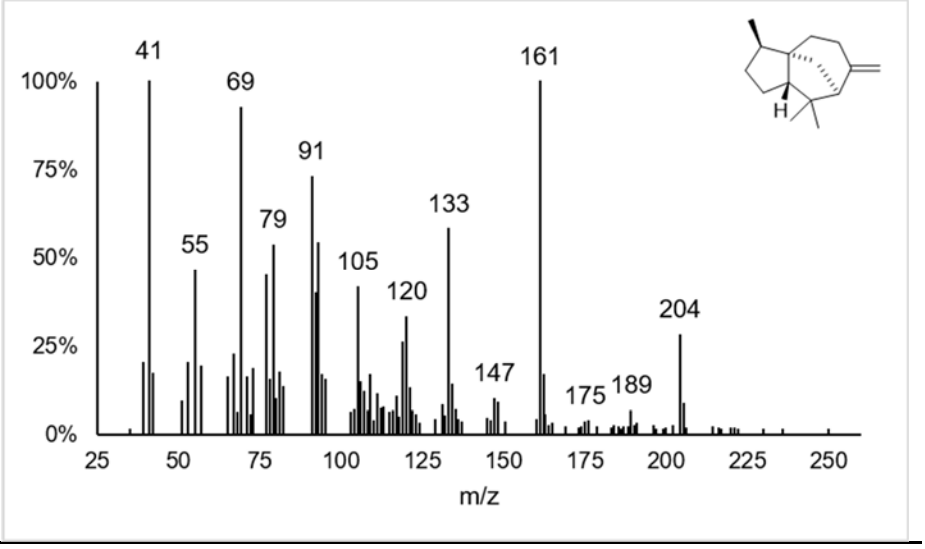

Table S2. *Cont.*

No.	Compound	Mass Spectrum
**6**	(*E*)-β-caryophyllene	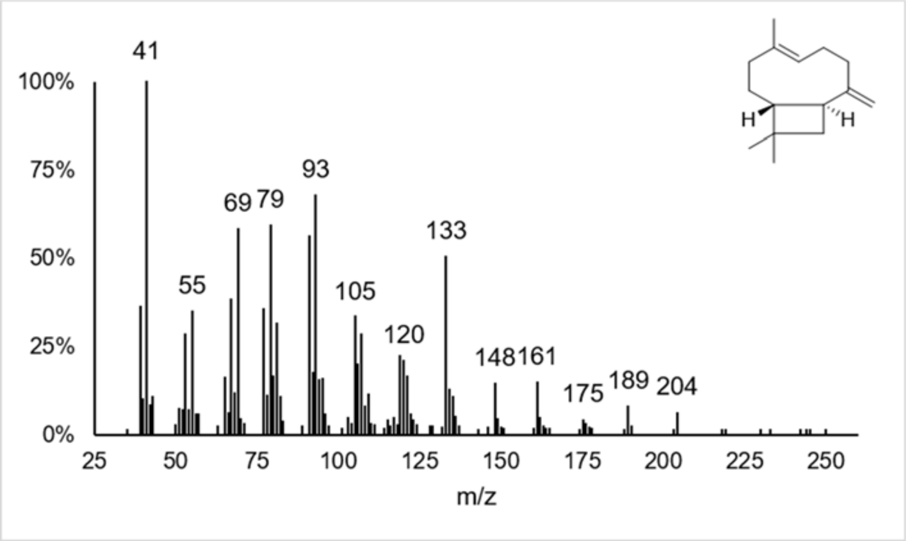
**14**	*cis*-thujopsene	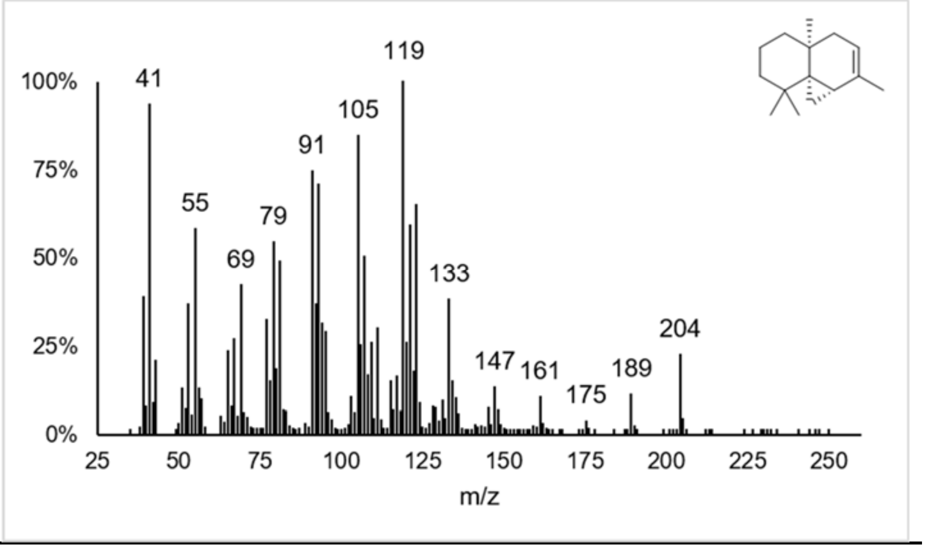

Table S2. *Cont.*

No.	Compound	Mass Spectrum
**2**	(*E*)-β-farnesene	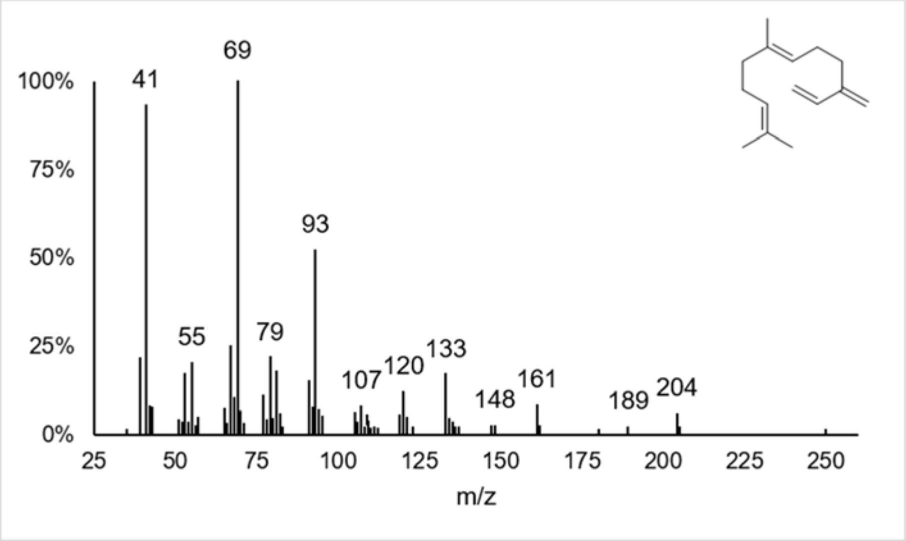
**16**	β-barbatene	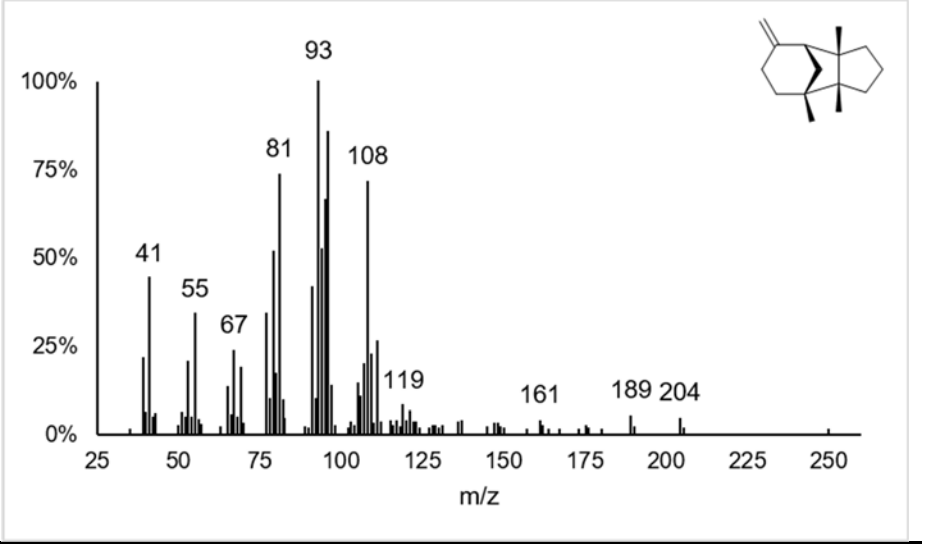

Table S2. *Cont.*

No.	Compound	Mass Spectrum
**10**	4-epi-α-acoradiene	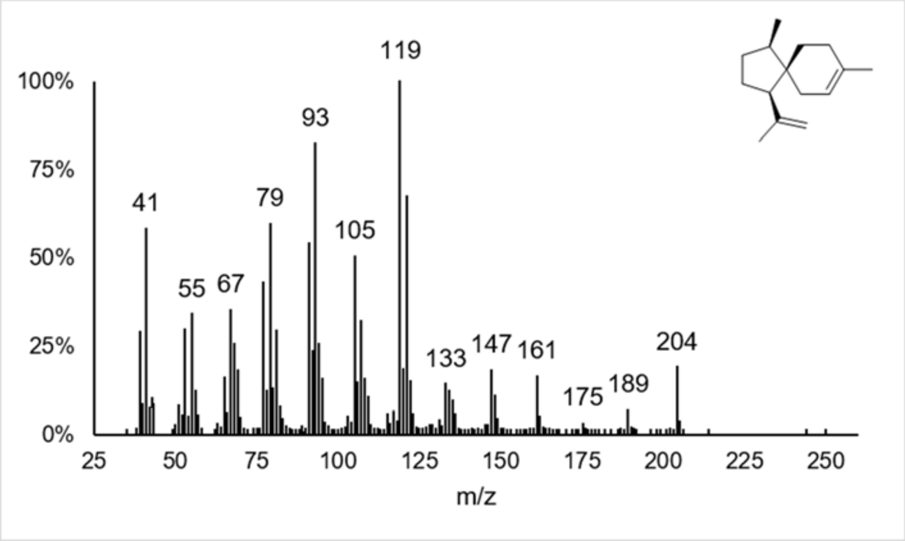
**9**	β-chamigrene	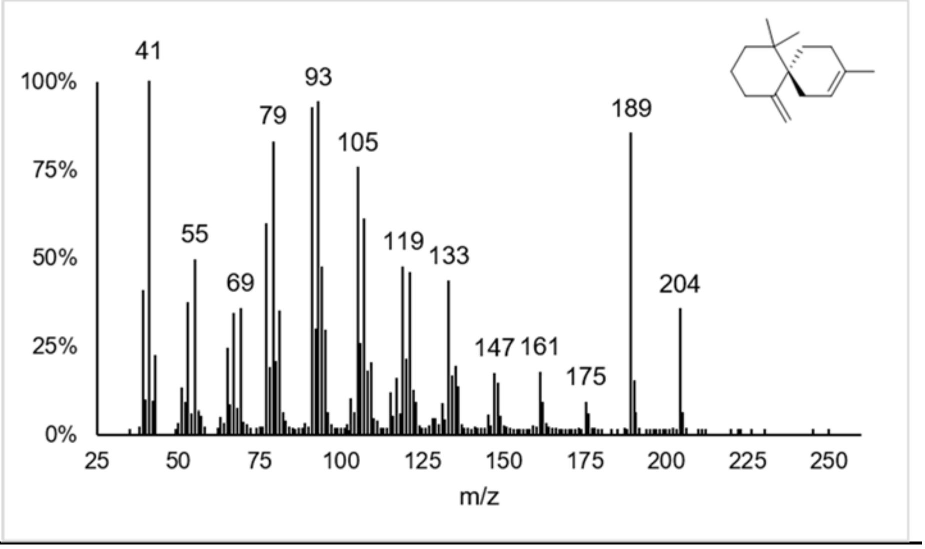

Table S2. *Cont.*

No.	Compound	Mass Spectrum
**3**	β-bisabolene	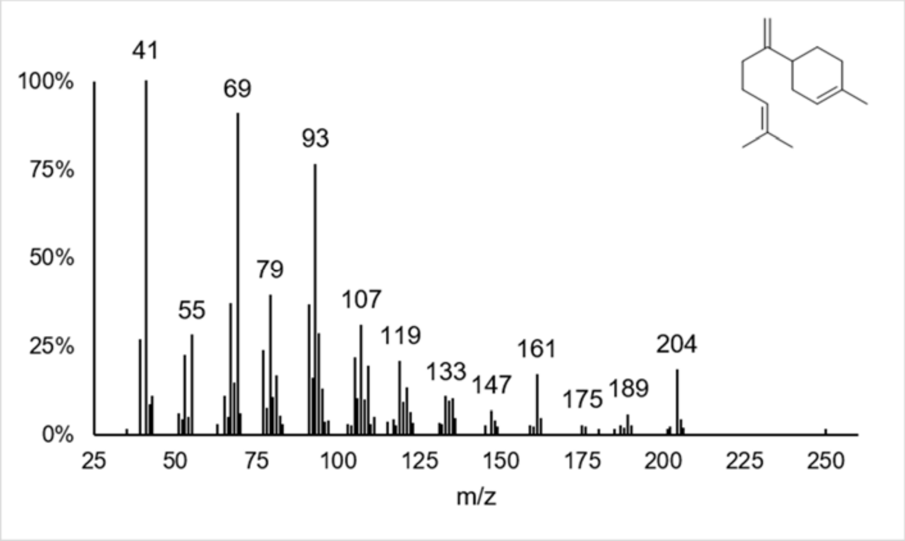
**1**	(*E*,*E*)-α-farnesene	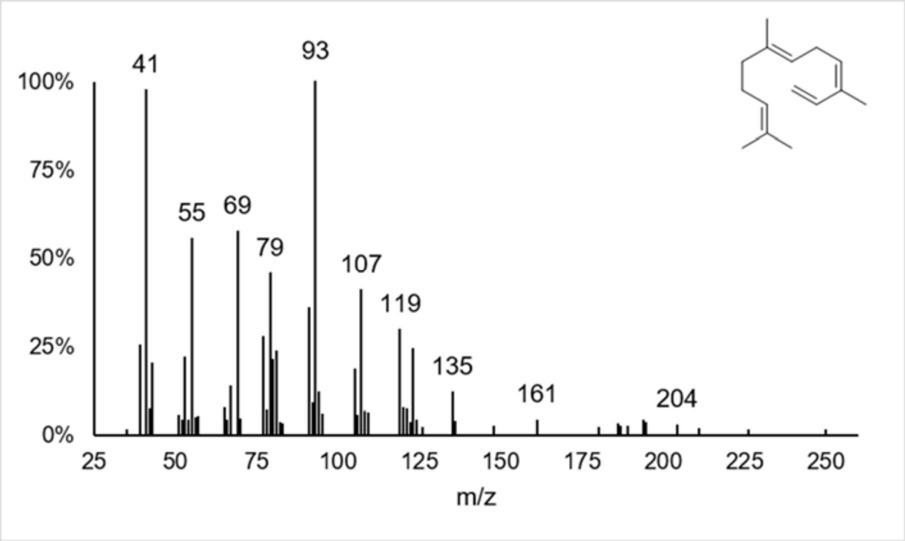

Table S2. *Cont.*

No.	Compound	Mass Spectrum
**8**	α-chamigrene	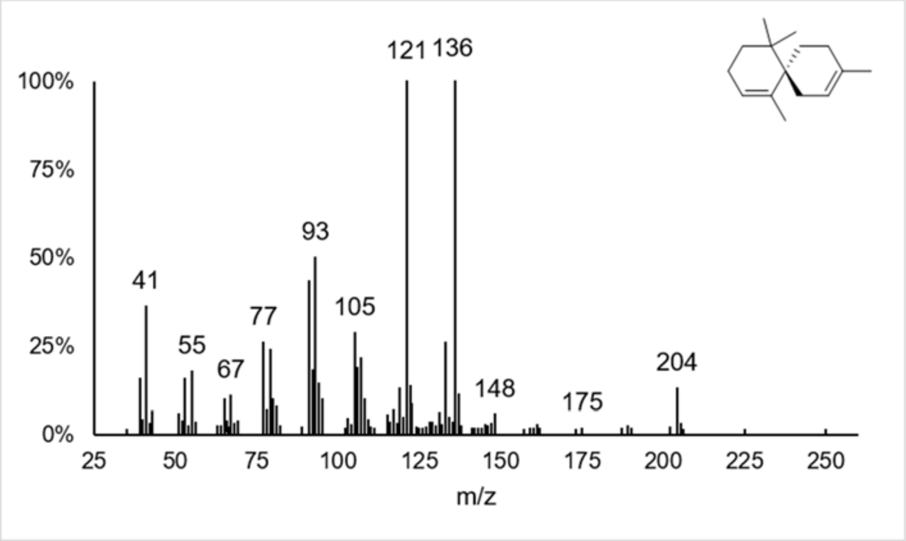
**4**	α-curcumene	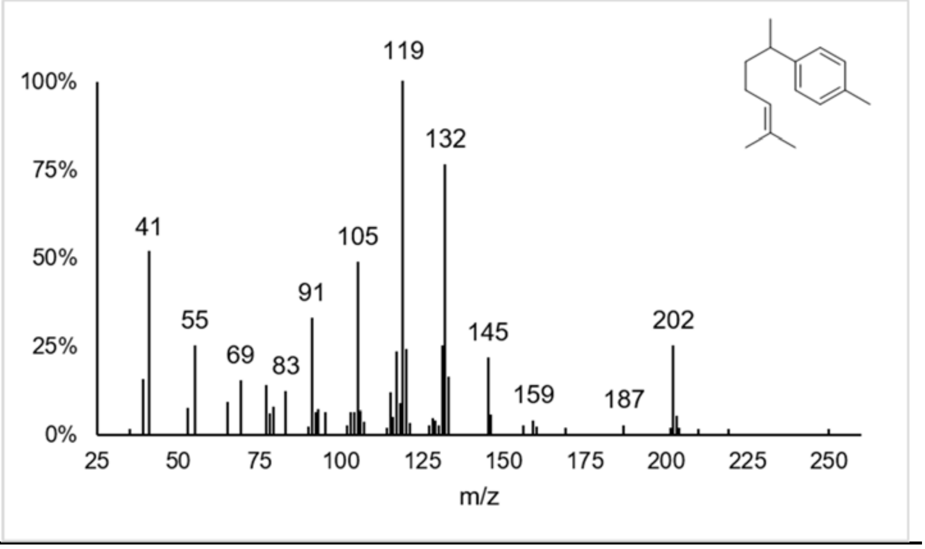

Table S2. *Cont.*

No.	Compound	Mass Spectrum
**7**	nootkatone	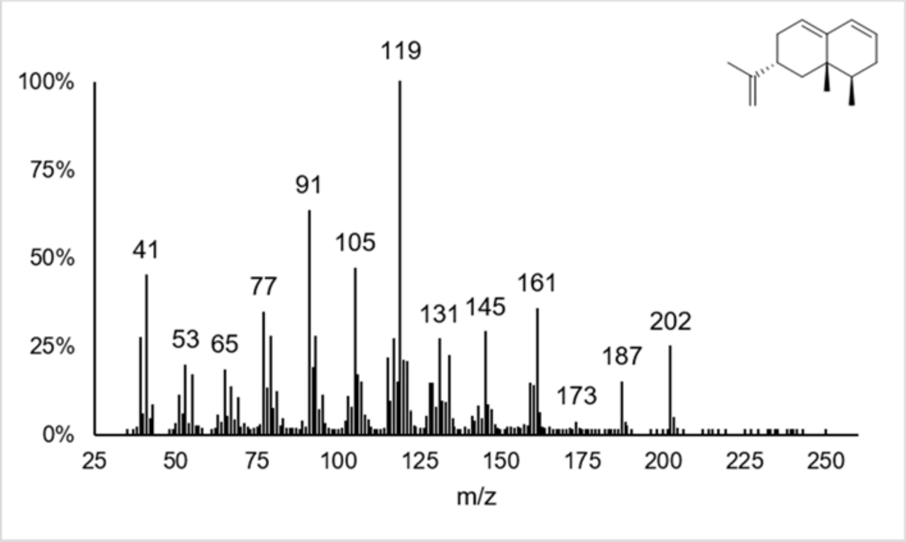
**11**	cuparene	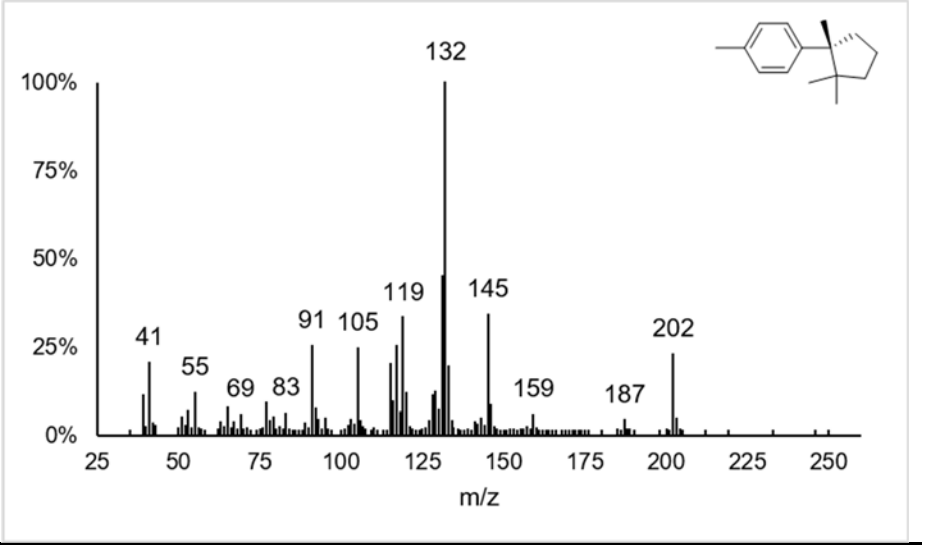
